# Associations between infant and maternal characteristics measured at child age 5 months and maternal feeding styles and practices up to child age two years

**DOI:** 10.1371/journal.pone.0261222

**Published:** 2022-01-07

**Authors:** Christine Helle, Elisabet R. Hillesund, Nina C. Øverby

**Affiliations:** Department of Nutrition and Public Health, Faculty of Health and Sport Sciences, University of Agder, Kristiansand, Norway; National Research Centre of Egypt, EGYPT

## Abstract

Facilitating positive feeding practices from infancy may be an important strategy to prevent childhood overweight and obesity. Since the feeding situation early in life constitutes a bidirectional relationship, it is important to understand the impact of both maternal and infant characteristics on maternal feeding practices to intervene in a customized and tailored way. Few studies have concurrently examined associations between maternal and infant characteristics in relation to early maternal feeding practices. The aim of the present study was to explore potential associations between infant and maternal characteristics measured at child age five months, and maternal feeding styles and practices during the child’s first two years. Cross-sectional data from a Norwegian randomized controlled trial in which participants responded to questionnaires at child age 5 months (n = 474), 12 months (n = 293) and 24 months (n = 185) were used to explore potential associations. All maternal and child predictor variables were collected at child age five months. Maternal feeding styles and practices were mapped using subscales from the *Infant Feeding Questionnaire* at child age 5 and 12 months and the *Child Feeding Questionnaire* and the *Parental Feeding Style Questionnaire* at child age 24 months. The subscale-scores were split into roughly equal tertiles, and the upper or lower tertile for the outcome of interest were used to create binary outcome variables. Multivariable binary logistic regression models were conducted for each outcome. We found that maternal education and mental health symptoms as well as infant weight, temperament and feeding mode were associated with maternal feeding styles and practices over time. Our findings indicate that risk factors which may have long-term implications for child weight and health outcomes can be identified early. Larger, population-based studies with a longitudinal design are needed to further explore these pathways.

## Introduction

The worldwide high prevalence of childhood overweight and obesity has directed attention to not only *what*, but also to *how* parents feed their children. The way parents feed their children influences the socialization and development of child eating behaviors, beginning as early as in infancy [1]. As eating behaviors and food preferences develop early in life, the child’s nutritional environment in these first years may have lifelong implications for weight and health outcomes [2]. In literature, parental feeding styles or beliefs describe the more general parent-child interactions across food-related situations whereas parental feeding practices refer to the food- or eating-specific behaviors or strategies that parents use to influence what, when and how much their children eat [3–5]. While some parental feeding styles and practices may facilitate the development of healthy eating behaviors, others may hinder such a development. Previous research has shown that parental feeding practices that are responsive to the child´s individual needs and cues of hunger and satiety, may initiate healthy eating patterns protective against obesity [6, 7]. In contrast, controlling or non-responsive feeding practices are characterized by a lack of reciprocity between the parent and child, have been associated with increased likelihood of developing overweight and obesity in children [8, 9]. A chronic mismatch of caregiver responsiveness to infant-feeding cues, such as feeding when the infant is not hungry, is hypothesized to have a role in the development of overweight by impairing the infant’s response to internal states of hunger and satiation [10].

Parental feeding practices in infancy have shown prospective associations with BMI in later childhood [11, 12]. Recently, a meta-analysis of four randomized controlled trials including more than 2000 mother-infant dyads, found that obesity prevention interventions commencing very early in childhood showed promising results regarding improved parental feeding practices and reduction in child BMI-for-age z-scores [13]. These findings, which if replicated on a wider scale, may have important public health implications. However, although facilitating positive feeding practices from infancy may be an important strategy to prevent childhood overweight and obesity, caution is needed when intervening in such a sensitive period. The feeding situation early in life constitutes a bidirectional relationship, where the behavior of parent and child vary in a give-and-take pattern. It is therefore necessary to understand the impact of both maternal and infant characteristics on maternal feeding practices to intercede in a customized and tailored way.

A systematic review from 2016 examined maternal correlates of maternal child feeding practices [14]. For lower socioeconomic background and psychopathology, they found significant relationships with increased maternal use of controlling feeding practices. Regarding maternal ethnicity and weight, findings were more inconsistent. The authors concluded that maternal correlates associated with maternal child feeding practices were complex, and that research examining the longitudinal pathways of these associations were urgent. Previous research has also shown evidence for child characteristics influencing maternal feeding behaviors. For toddlers, maternal use of controlling feeding practices has shown to be associated with the child being a daughter [15, 16]. In infancy, studies have revealed that boys tend to be introduced to solid food earlier than girls [17–19]. In addition, both infant weight [4, 20, 21] and temperament [22] have been associated with maternal use of feeding practices that may influence risk of developing childhood overweight [22–24].

So far, few studies have concurrently examined associations between maternal- and infant characteristics in relation to maternal feeding practices in infancy. Despite the acknowledged importance of investigating dyadic parent-child interactions regarding feeding, the vast majority on this field has adopted a uni-directional perspective [25]. Hence, the relative and unique contribution of maternal and infant correlates to maternal feeding practices in early life remains unclear [26]. Even fewer studies have examined whether maternal and child characteristics during infancy may be associated with maternal use of feeding practices over time. More knowledge about infant and maternal factors that may influence maternal use of nonresponsive feeding practices is important in clinical practice, where available resources and time for guidance may be limited and priorities necessary. Therefore, the aim of the study was to explore longitudinal associations between infant and maternal characteristics measured at child age five months, and maternal feeding styles and practices in a sample of Norwegian mother-child dyads followed up at child age 5, 12 and 24 months.

## Materials and methods

### Study design and participants

This study presents a secondary analysis of data from the Norwegian randomized controlled trial *Early Food for Future Health*. This is a primary prevention intervention, commenced when children were between 6 and 12 months of age, aiming to promote healthy food habits from infancy [27]. In 2016, parents from all over Norway were eligible to participate in this study if they had a 3–5 months old infant born after gestational week 38, mastered Norwegian and were responsible for providing food to their infant. In 2016, a total of 715 mothers completed the baseline questionnaire at child age 5 months (T1). Post-intervention data were collected at child age 12 months (T2), and follow-up data at child age 24 months (T3).

All data were collected from a web-based, self-administered questionnaire. At baseline, it was obligatory to respond to all questions with an exception for child anthropometric data. Infant weight and length at child age five months were measured at the child health clinics and reported by the mothers. If the child had not yet been to the clinic at this time-point, mothers had the opportunity to omit responding. For the current paper, we only included the participants who had reported on infant weight and length. This resulted in n = 474 at child age 5 months (T1), n = 293 at child-age 12 months (T2) and n = 185 at child age 24 months (T3). The baseline characteristic of participants excluded from the current study due to missing child anthropometric data did not differ from those included in the study, except for the infants being one week younger (21.5 weeks vs. 22.6 weeks respectively). The proportions of study participants excluded from analyses due to missing baseline child anthropometric data were 34% at T1 (n_tot_ = 715), 36% at T2 (n_tot_ = 455) and 37% at T3(n_tot_ = 295).

Informed consent from all participating parents were obtained digitally upon registration on the study´s homepage. The Norwegian Centre for Research Data evaluated and approved the study (http://pvo.nsd.no/prosjekt/43975). The clinical trial registration number for the original trial is ISRCTN13601567, results from the original study have previously been published elsewhere [28, 29].

### Measures

#### Feeding practices and feeding styles

Maternal feeding practices and beliefs at child age 5 and 12 months were assessed by *The Infant Feeding Questionnaire* (IFQ) [30]. The original validated seven-factor questionnaire contains 20 items, and retrospectively measures feeding practices and beliefs during the first year of life. Responses were given on a five-point Likert Scale ranging from “never” to “always” or from “disagree a lot” to “agree a lot”. For this study, we used the version included in the NOURISH-study [31]. This is a five-factor questionnaire, where the tense is changed from past to present to accommodate concurrent use. This modified version has previously been used in several studies [24, 32, 33]. A translation-back-translation procedure was applied by two different investigators to obtain a Norwegian version. The English translation was compared with the original IFQ, and minor discrepancies were revised based on consensus. In the original study, a confirmatory factor analysis was performed to determine the fit of the five-factor structured IFQ applied to our study-population. This resulted in the same five factors as in the Australian version [27]. For the present study, Cronbach´s α for the four IFQ- subscales *Concern about underweight*, *Concern about overweight*, *Awareness of hunger and satiety cues* and *Using food to calm* at child age 5 and 12 months ranged from 0.6–0.8, indicating a moderate to good internal consistency. For the subscale *Feeding on a schedule*, Cronbach´s α was low (0.5), and this factor was excluded from further analyses.

At child age 24 months, maternal feeding practices were assessed by *The Child Feeding Questionnaire* (CFQ) developed by Birch et.al. [34]. This is one of the most widely used questionnaires to assess parental feeding practices and examines parental attitudes and behaviors towards children´s diet. We included two subscales, *Restriction* (attempting to control children’s diet by restricting access to unhealthy foods) and *Pressure* (pushing children to eat more food or to increase their intake of healthy foods), to assess maternal controlling feeding practices in our sample. The CFQ was previously translated to Norwegian, and the same subscales were used in the Norwegian population-based MoBa study [35]. Cronbach´s α for the *Restriction* and *Pressure* subscales was 0.66 and 0.63 respectively, indicating a moderate internal consistency for these subscales.

In addition, we used *The Parental Feeding Style Questionnaire* (PFSQ) designed by Wardle et al. [36], a tool for assessing four aspects of parental feeding styles; *Instrumental feeding*, *Encouragement*, *Emotional feeding* and *Control over eating*. PFSQ is a widely used questionnaire which shows good test-retest reliability, is validated in several cultures [37], and has previously been used in Norway [38]. For the four PFSQ-subscales, Cronbach´s α varied between 0.7 and 0.8, indicating a moderate to good internal consistency.

#### Maternal and infant characteristics

All demographic data were obtained from the baseline questionnaire of the original study *Early Food for Future Health and* collected between March and September 2016. We included a broad range of maternal and infant characteristics known from earlier studies to influence early feeding.

*Maternal characteristics*. Maternal age was computed based on self-reported birth date. Maternal education was originally mapped by asking “What is your highest completed education” with response-options on a 7-category scale. These were subsequently recoded into *high* vs *low* education *(college/university vs*. *no college/university*). Maternal BMI was calculated based on self-reported weight and height at inclusion, five months post-partum.

Maternal symptoms of depression and anxiety were assessed with the short version (SCL 8) of the Hopkins Symptoms Checklist (SCL 90). This is a well-established psychometric instrument [39], and the short version used in this study has previously been used in the Norwegian population-based MoBa study [40]. Symptoms are mapped as “Have you been bothered by any of the following during the last two weeks?” with four response-categories ranging from *not bothered* to *very bothered*. These response-categories are rated from 1 to 4, with higher scores reflecting more severe symptoms. The SCL 8 total score was computed by adding the eight item scores and dividing the sum-score on the number of items. Cronbach´s alpha for this scale in our sample was 0,83, indicating a good internal consistency.

*Infant characteristics*. Infant gender was self-reported by the mothers. Infant BMI-for-age z-scores at child age 5 months were computed based on the mothers self-reported measures taken at the child health center on weight, length and date of visit at the child health center. The mothers had the opportunity to omit responding if the child had not visited the child health center at child age 5 months. The BMI-for-age z-scores, adjusted for age and gender, were calculated using the software program WHO Anthro version 3.2.2. (Department of Nutrition, WHO, Geneva, Switzerland) [41].

Infant temperament was assessed with a short version of the fussy/difficult subscale from *The Infant Characteristics Questionnaire–* 6 months form (ICQ-6) [42]. This seven-items version of the questionnaire has also previously been used in the Norwegian population-based MoBa study [43]. Mothers were asked to report to which extent they agree with statements concerning their infant´s temperament on a 7-point scale ranging from *Completely disagree* to *Agree completely*. Higher scores indicate perceived greater infant difficulties. An average score based on the seven items was calculated. Cronbach´s alpha for this scale was 0,72, indicating a moderate internal consistency.

At five months of age, the mothers were asked “*How often does the child have breast milk nowadays*?*”* with 8 answer-options from *never* to *5 times a day or more*. Breastfeeding status was dichotomized as *Breastfeeding* vs. *No breastfeeding*.

### Statistical analysis

Tests for normality were conducted for each of the four included IFQ-subscales. Only the subscale *Using food to calm* was found to be normally distributed. We therefore used binary logistic regression to explore potential associations between maternal and infant characteristics and maternal feeding practices and beliefs. For the four IFQ factors, the sample was split into three roughly equal groups using the tertile cutoff-points for the factor scores. The upper tertile was separated from the other two tertiles to create outcome binary variables for *Concern about underweight*, *Concern about overweight* and *Using food to calm*. For the factor *Awareness of infant hunger and satiety cues*, the lower tertile was separated from the other two tertiles to create the outcome variables. This method is in line with what have been previously published [11, 24].

For the *Child Feeding Questionnaire*, only the subscale *Restriction* was found to be normally distributed. For the *Parental Feeding Style Questionnaire*, only the subscale *Control* was normally distributed. We decided to use the same procedure for these instruments as for the IFQ by splitting each of the factors into equal tertiles. We created six binary outcome-variables using the upper-tertile as a high score for the CFQ`s *Pressure* and *Restriction* and for the PFSQ`s *Control*, *Emotional Feeding* and *Instrumental Feeding*, and the lower tertile for a low score on *Encouragement*.

A total of 14 different multivariable binary logistic regression models were conducted, one for each of the four IFQ factors at child age 5 and 12 months respectively and 6 for the different CFQ and PFSQ factors at child age 24 months. We used a complete case approach, and all predictor variables were collected at child age five months. The following maternal variables were included: Age, education, BMI and symptoms of depression/anxiety. The included infant variables were: Gender, BMI-for-age z-score at child age 5 months, temperament and milk-feeding mode.

Potential violations of multicollinearity assumptions were tested by treating the categorical values as continuous in a linear regression and collinearity diagnostics were performed. There was no multicollinearity between the variables in the logistic regression models. The infant and maternal predictor variables above were initially explored in univariate tests in all the different regression models. All predictor variables associated with one or more of the feeding factor outcomes with a p-value ≤ 0.2 and were included. The predictor variables were included simultaneously and mutually adjusted in the regression models for each feeding practice factor. This method is in line with previous research [44]. Lastly, as a post-hoc analysis we explored the relationship between maternal mental health and infant temperament to look for potential interaction-effects. We used centered predictor variables for maternal mental health and infant temperament to create an interaction-term, all the analyses were run with the interaction-term added. The study size was calculated according to the original randomized controlled trial [27]. Sample size estimates for the original trial´s primary outcomes are previously published [28]. Statistical analysis was conducted using SPSS 25.0 (IBM Corp., Somers; NY, USA). Statistical significance was defined as a p-value less than 0.05.

## Results

The characteristics of all the participating mothers and infants at child age five months are shown in Table 1. Maternal age ranged from 18 to 44 years (mean 30.3). A high proportion of the mothers had higher education (81.6%) compared to national data for women in the same age group [45]. Mean maternal BMI five months post-partum was 24.8 kg/m^2^. The mean SCL 8 score in this sample was 1.25 (possible range 1.00–4.00), which is similar to pre-pregnancy and pregnancy mean scores found in previous Norwegian population-based studies [46, 47]. The infants´ mean age was 21.5 weeks (4.9 months), the proportion of boys was 52% and close to the national average [48]. The mean infant BMI-for-age z-score was 0.02, indicating a mean child BMI close to the average for infants at child age five months. The infant mean-score on the fuzzy/difficult subscale from the Infant characteristics’ questionnaire ICQ-6 was 2.50 (possible range 1.00–4.00). A total of 86% of the infants were partially or fully breastfed at child age 5 months. This is somewhat higher than the results from the latest national survey from 2020 (78% at child age 6 months) [49].

**Table 1:** Characteristics of the participating mother-infant dyads at child age 5 months (n = 474).

Characteristics		Values	Mean (SD) or %*
**Mothers**			
	Age	Years	30.3 (4.4)
	University education	Yes	81.6
	Maternal BMI at child age 5 months	kg/m^2^	24.8 (4.1)
	Maternal depression/anxiety-score at child age 5 months	SCL 8-score	1.25 (0.34)
**Infants**			
	Age	Weeks	22.6 (1.5)
	Gender	Boy	51.9
	BMI-for-age z-score at child age 5 months	kg/m^2^	0.02 (0.98)
	Infant temperament score at child age 5 months	ICQ fuzzy-diff. score	2.50 (0.87)
	Milk feeding mode at child age 5 months	Breast feeding	86.1

* Valid percentages for categorical variables and means with SD for continuous variables.

Based on status at T3, the non-completer mothers were less likely to have a university education (77.5% vs. 88.1%) compared to mothers who completed at T3, and their infants were slightly older (22.7 vs. 22.3 weeks). There were no differences between completers and non-completers in terms of maternal age, weight-status, symptoms of anxiety/depression, infant gender, BMI-for-age z-score, temperament and feeding mode at child age 5 months.

The cross-sectional associations between maternal and infant characteristics and maternal feeding practices and beliefs at child age five months are presented in Table 2. Mothers with a higher BMI were marginally more likely to have a high score on both *Concerned about underweight* (OR = 1.06; p = 0.028) and *Concern about overweight* (OR = 1.06; p = 0.030). Mothers with more symptoms of anxiety/depression were more likely to have a high score on *Concerned about overweight* (OR = 2.23; p = 0.012). For infant characteristics, infants with a higher BMI-for-age z-score were less likely to have mothers with a high score on *Concern about underweight* (OR = 0.44; p<0.001), and more likely to have mothers with a high score on *Concern about overweight* (OR = 1.55; p<0.001). There was a clear association between child temperament and infant feeding practices and beliefs. Infants with high scores (indicating more fussy/difficult temperament) were more likely to have mothers with high scores on both *Concern about underweight* (OR = 1.59; p<0.001), *Concern about overweight* (OR = 1.66; p<0.001) and *Using food to calm* (OR = 1.72; p<0.001). In addition, infant with high scores on temperament were more likely to have mothers with a low score on *Awareness of infant hunger and satiety cues* (OR = 1.90; p<0.001). For feeding mode, we found that infants who were not breastfed were more likely to have mothers with a high score on *Using food to calm* compared to infants who were breastfed (OR = 5.56; p<0.001).

**Table 2:** Associations between maternal and child covariates and maternal feeding beliefs and practices at child age five months estimated by multivariable logistic regression.

Characteristics			Infant feeding practices and beliefs 5 months n = 474											
			*Concern about underweight High score* n = 127 (27%)			*Concern about overweight High score* n = 136 (29%)			*Awareness hunger and satiety cues Low sore* n = 203 (43%)			*Using food to calm High score* n = 153 (32%)		
**Maternal**			OR	95% CI	P-value	OR	95% CI	P-value	OR	95% CI	P-value	OR	95% CI	P-value
	Age (years)		0.992	0.941–1.047	0.777	1.004	0.953–1.058	0.874	0.977	0.931–1.024	0.327	0.976	0.928–1.028	0.360
	Education													
		High (n = 387)	1.000			1.000			1.000			1.000		
		Low (n = 87)	1.107	0.593–2.065	0.749	0.945	0.524–1.706	0.852	1.710	0.969–3.015	0.064	1.147	0.632–2.082	0.653
	BMI (kg/m^2^)		1.066	1.008–1.127	**0.025**	1.060	1.006–1.117	**0.030**	0.985	0.937–1.035	0.542	1.009	0.957–1.063	0.743
	Depression/anxiety (SCL-8 score)		1.121	0.590–2.131	0.726	2.230	1.192–4.169	**0.012**	1.392	0.771–2.513	0.272	1.286	0.696–2.376	0.421
**Infant**														
	Gender													
		Girl (n = 228)	1.000			1.000			1.000			1.000		
		Boy (n = 246)	1.046	0.670–1.632	0.843	1.113	0.727–1.705	0.622	0.819	0.556–1.207	0.313	1.409	0.934–2.126	0.103
	BMI-for-age z-score		0.435	0.334–0.568	**<0.001**	1.553	1.245–1.937	**<0.001**	0.819	0.669–1.001	0.051	1.108	0.900–1.365	0.334
	Temperament (ICQ fuzzy-diff. score)		1.588	1.235–2.042	**<0.001**	1.652	1.290–2.116	**<0.001**	1.855	1.466–2.347	**<0.001**	1.723	1.352–2.196	**<0.001**
	Feeding mode													
		Breast-feeding (n = 408)	1.000			1.000			1.000			1.000		
		No breast-feeding (n = 66)	0.935	0.499–1.784	0.839	1.252	0.663–2.363	0.488	0.813	0.454–1.455	0.486	5.559	2.370–13.044	**<0.001**

The associations between infant and maternal characteristics at child age 5 months and maternal feeding practices and beliefs at child age 12 months are presented in Table 3. Mothers with lower education were more likely to have a high score on *Concern about overweight* (OR = 2.93; p = 0.027) and a low score on *Awareness hunger and satiety cues* (OR = 2.23, p = 0.042) compared to mothers with higher education. In line with results from five months, infants with higher BMI-for-age-z-scores at child age 5 months were less likely to have mothers with a high score on *Concern about underweight* (OR = 0.64; p = 0.004) and more likely to have mothers with a high score on *Concern about overweight* (OR = 1.39; p = 0.029) at child age 12 months. Infants with higher BMI-for-age-z-scores at child age 5 months were further less likely to have mothers with a low score on *Awareness hunger and satiety cues* (OR = 0.70; p = 0.008). Also, infants with more fussy/difficult temperament at child age 5 months were more likely to have mothers with a low score on *Awareness hunger and satiety cues* (OR = 1.50; p = 0.008) at child age 12 months. For feeding mode, infants who were not breastfed were less likely to have mothers with a high score on *Concern about underweight* (OR = 0.42; p = 0.034).

**Table 3:** Associations between maternal and child covariates at child age 5 months and maternal feeding beliefs and practices at child age 12 months estimated by multivariable logistic regression.

Characteristics			Infant feeding practices and beliefs 12 months n = 293											
			*Concern about underweight High score* n = 78 (27%)			*Concern about overweight High score* n = 77 (26%)			*Awareness hunger and satiety cues Low score* n = 146 (50%)			*Using food to calm High score* n = 120 (41%)		
**Maternal**			OR	95% CI	P-value	OR	95% CI	P-value	OR	95% CI	P-value	OR	95% CI	P-value
	Age (years)		1.034	0,968–1.105	0.321	0.984	0.919–1.053	0.640	0.969	0.912–1.028	0.297	0.959	0.903–1.019	0.174
	Education													
		High (n = 252)	1.000			1.000			1.000			1.000		
		Low (n = 41)	1.322	0.548–3.186	0.535	2.930	1.128–7.611	**0.027**	2.228	1.032–4.811	**0.041**	1.099	0.525–2.300	0.801
	BMI (kg/m^2^)		0.963	0.899–1.032	0.290	1.065	0.997–1.37	0.061	1.001	0.942–1.064	0.982	0.979	0.921–1.040	0.487
	Depression/anxiety (SCL8-score)		1.174	0.499–2.763	0.713	1.928	0.845–4.398	0.119	0.939	0.438–2.013	0.872	1.844	0.854–3.983	0.119
**Infant**														
	Gender													
		Girl (n = 141)	1.000			1.000			1.000			1.000		
		Boy (n = 152)	0.795	0.458–1.378	0.414	0.739	0.424–1.290	0.288	1.110	0.681–1.810	0.674	0.669	0.410–1.092	0.108
	BMI-for-age z-score		0.646	0.478–0.873	**0.004**	1.403	1.051–1.872	**0.021**	0.696	0.534–0.906	**0.007**	0.944	0.733–1.216	0.658
	Temperament (ICQ fuzzy-diff. score)		1.116	0.805–1.546	0.511	1.153	0.833–1.595	0.390	1.496	1.110–2.018	**0.008**	1.298	0.969–1.738	0.080
	Feeding mode													
		Breastfeeding (n = 261)	1.000			1.000			1.000			1.000		
		No breastfeeding (n = 32)	0.414	0.185–0.925	**0.032**	0.734	0.320–1.684	0.466	1.507	0.686–3.311	0.307	0.851	0.391–1.852	0.685

The associations between infant and maternal characteristics at child age 5 months and maternal feeding practices assessed by the Child Feeding Questionnaire at child age 24 months are presented in Table 4. We found that mothers with more symptoms of anxiety and depression in the postnatal period were more likely to have a high score on the subscale *Pressure* (OR = 3.48; p = 0.026) at child age 24 months. We also found that infants with higher BMI-for-age z-score at five months of age were less likely to have a mother with a high score on *Pressure* at child age 24 months (OR = 0.64; p = 0.019).

**Table 4:** Associations between maternal and child covariates at child age 5 months and maternal use of controlling feeding practices at child age 24 months estimated by multivariable logistic regression.

Characteristics			*The Child Feeding Questionnaire*, *24 months* n = 185					
			*Pressure High score* n = 55 (30%)			*Restriction High score* n = 43 (23%)		
**Maternal**			OR	95% CI	P-value	OR	95% CI	P-value
	Age (years)		1.005	0.929–1.088	0.895	1.001	0.919–1.090	0.985
	Education							
		high (n = 163)	1.000			1.000		
		low (n = 22)	1.658	0.556–4.942	0.365	1.250	0.395–3.961	0.704
	BMI (kg/m^2^)		1.051	0.965–1.144	0.252	0.999	0.910–1.096	0.980
	Depression/anxiety (SCL8-score)		3.394	1.145–10.062	**0.028**	2.014	0.676–6.007	0.209
**Infant**								
	Gender							
		Girl (n = 91)	1.000			1.000		
		Boy (n = 94)	0.555	0.273–1.132	0.105	1.093	0.522–2.291	0.814
	BMI-for-age z-score		0.652	0.454–0.936	**0.021**	0.780	0.533–1.140	0.199
	Temperament (ICQ fuzzy-diff. score)		0.883	0.589–1.321	0.544	1.346	0.883–2.051	0.167
	Feeding mode							
		Breastfeeding (n = 166)	1.000			1.000		
		No breastfeeding (n = 19)	0.565	0.199–1.608	0.285	3.231	0.679–15.380	0.141

For the Parental Feeding Style Questionnaire presented in Table 5, we found that mothers with a higher anxiety/depression-score in the postnatal period were more likely to have a high score on both *Instrumental Feeding* (OR = 5.58; p = 0.004) and *Emotional Feeding* (OR = 3.74; p = 0.024) at child age 24 months. For feeding mode, we found that mothers of infants who were not breastfed at child age five months were more likely to have a high score on *Emotional Feeding* at child age 24 months (OR = 4.79; p = 0.033).

**Table 5:** Associations between maternal and child covariates at child age 5 months and maternal feeding styles at child age 24 months estimated by multivariable logistic regression.

Characteristics			*The Parental Feeding Style Questionnaire*, *24 months n = 185*											
			*Encouragement Low score* n = 79 (43%)			*Instrumental Feeding High score* n = 51 (28%)			*Control High score* n = 58 (31%)			*Emotional Feeding High score* n = 60 (32%)		
Maternal			OR	95% CI	P-value	OR	95% CI	P-value	OR	95% CI	P-value	OR	95% CI	P-value
	Age (years)		1.075	0.999–1.157	0.054	1.064	0.981–1.154	0.137	0.978	0.902–1.060	0.585	1.067	0.985–1.155	0.111
	Education													
		High (n = 163)	1.000			1.000			1.000			1.000		
		Low (n = 22)	1.410	0.507–3.918	0.510	0.993	0.341–2.895	0.990	1.067	0.367–2.954	0.940	1.051	0.372–2.971	0.926
	BMI (kg/m^2^)		1.022	0.945–1.105	0.584	1.025	0.939–1,120	0.578	1.041	0,955–1.135	0.361	1.073	0.985–1.169	0.109
	Depression/anxiety (SCL 8-score)		0.592	0.215–1.636	0.312	5.246	1.654–16.637	**0.005**	0.564	0.176–1.809	0.336	3.768	1.201–11.820	**0.023**
Infant														
	Gender													
		Girl (n = 91)	1.000			1.000			1.000			1.000		
		Boy (n = 94)	1.115	0.596–2.087	0.733	0.741	0.361–1.520	0.413	1.277	0.644–2,533	0.484	0.898	0.453–1.782	0.758
	BMI-for-age z-score		0.940	0.686–1.288	0.700	0.913	0.636–1.311	0.622	0.868	0.614–1.227	0.422	1.160	0.818–1.645	0.406
	Temperament (ICQ fuzzy-diff. score)		1.260	0.876–1.812	0.212	1.220	0.811–1.837	0.339	0.731	0.486–1.101	0.134	1.460	0.984–2.167	0.060
	Feeding mode													
		Breastfeeding (n = 166)	1.000			1.000			1.000			1.000		
		No breastfeeding (n = 19)	1.134	0.418–3.078	0.805	2.980	0.733–12.115	0.127	0.695	0.246–1.959	0.491	4.781	1.135–20.144	**0.033**

As maternal mental health and infant temperament appeared to be important explanatory variables, we wanted to explore the relationship between these two variables further and performed a post-hoc analysis testing for potential interaction effects. We found a significant interaction-term for *Using food to calm* at child age 5 months (p = 0.011). We explored this further by splitting the study population by the mean values for infant temperament (ICQ-score) and maternal symptoms of anxiety/depression (SCL8-score) in a high- and low group. In the group with low score on infant temperament, mothers with higher levels of depression and anxiety were significantly more likely to have a high score on *Using food to calm* (OR 3.17; p = 0.016). When looking at the group with a high score on infant temperament or the complete study-sample, this was not the case.

## Discussion

The aim of this study was to explore potential associations between infant and maternal characteristics measured at child age 5 months and maternal feeding styles and practices over the child’s first two years by following a sample of Norwegian mother-child dyads at child age 5, 12 and 24 months. Our main findings will be discussed below in light of previous research and their implications for public health nutrition action and future research.

### Maternal characteristics

#### Maternal socio-demographic factors

A review exploring the relationship between socioeconomic position and the early-life predictors of obesity, concluded that a strong socioeconomic gradient exists for the majority of early-life predictors of obesity [50]. Maternal demographic factors such as age, education and socioeconomic status have in previous research shown to influence both early child feeding behaviors like duration of breastfeeding, early weaning and child consumption of fruit and vegetables [51–56] as well as early maternal feeding practices [14, 26]. Encouragement and support of healthy feeding practices are especially important for socioeconomically disadvantaged mothers, as their infants are at increased risk of early childhood obesity [57].

In our study we found a significant association between maternal education and maternal feeding practices at child age 12 months, where mothers without a university degree reported lower awareness of the child’s hunger and satiety cues. At child age 5 months the same association was almost significant (p = 0.06). Previous studies examining this relationship by the IFQ have not revealed the same association [24, 26, 44]. However, a 2014 systematic review of maternal correlates of maternal feeding practices found higher maternal education to be associated with lower use of controlling feeding practices like restriction and pressure and lower maternal education related to use of food as incentive [14]. These types of controlling feeding practices may to a lesser extent be captured during infancy by the Infant Feeding Questionnaire. Although we found no associations between maternal education and use of restriction and pressure measured by the CFQ at child age 24 months, our results could indicate an association between lower maternal education and less responsive feeding practices in infancy through lower awareness of infant hunger and satiety cues.

We found no associations between maternal age and maternal feeding practices as measured by the *Infant Feeding Questionnaire* (IFQ). This is congruent with previous research employing the IFQ [24, 26, 44].

#### Maternal mental health

A growing body of research points to common maternal mental disorders such as depression and anxiety as risk factors for impaired child development, including associations with preterm birth, low birth weight, and poor infant growth and cognitive development [58, 59]. Potential mechanisms may be related to less maternal sensitivity or responsiveness affecting the interplay between mother and child. This is associated with higher rates of negative emotional expressions and less predictable and consistent parenting [60]. Literature has also emphasized the importance of timing of maternal depression. Depressive symptoms in the postnatal period occur in a sensitive period of child development. The infant may therefore be more susceptible to the effects of maternal depression, which may have long-term consequences and persist into adolescence [61].

Maternal symptoms of anxiety and depression have in previous research been associated with a lower extent of breast feeding [46], a less wholesome and a more unhealthy child diet [62], and increased use of control and pressure to eat [46, 63–65]. We found that maternal mental health symptoms of depression and anxiety in early infancy was associated with maternal feeding style and practices up to child age two years. At child age 24 months, mothers who reported more symptoms of anxiety and depression at child age 5 months were more likely to use controlling types of feeding styles and practices measured by the factors *Instrumental feeding* (PFSQ) and *Pressure* (CFQ) and to have a high score on the factor *Emotional feeding* (PFSQ). We also found that mothers with higher symptom-scores of depression and anxiety were more likely to use food to calm at child age 5 months when the child had a mild or normal temper. Mental health problems may lower the threshold for coping with the child’s negative emotions and more easily activate own feelings of stress and worry. Using food to calm or soothe the child may therefore be an understandable strategy. Maternal negative affect has in previous research been associated with emotional feeding practices [66]. It has further been found that emotional child-feeding practices are positively associated with obesogenic child eating behaviors like higher levels of emotional eating in children [38, 67]. Our findings indicate that it may be important to support mothers with depression or anxiety in how to deal with negative child affect in non-food oriented ways independent of perceived child temperament.

The relationship between maternal psychopathology and maternal feeding styles and practices have previously been assessed in several studies [62, 63, 68–70] and systematic reviews [71, 72]. Our results are in concordance with this research, suggesting that maternal mental health issues may interfere with the ability to promote a healthier feeding environment with positive social interactions. We found that maternal symptoms of depression and anxiety in the postnatal period were associated with lower responsiveness in feeding at child age 2 years. Although our findings could be explained by maternal mental health being stable over time, our study contributes to previous research by confirming the potential long-term impact of maternal postnatal mental health symptoms on child feeding styles and practices.

#### Maternal weight

Maternal weight is a strong predictor for childhood obesity [50, 73]. However, the relationship between maternal weight and maternal feeding practices is more unclear. A systematic review of maternal correlates of child feeding practices including seven studies exploring this combination, concluded that the findings were inconsistent [14].

We found that higher maternal body mass index (BMI) predicted slightly more concern about their infants being under- and overweight at child age 5 months. Nevertheless, the odds-ratio was 1.06 and the clinical importance of these results may therefore be without significance. At child age 12 and 24 months, we found no associations between maternal weight-status measured at child age 5 months and subsequent maternal feeding styles or practices.

Our findings replicate previous research. A previous study from Australia examining the relationship between maternal pre-pregnancy weight status and maternal feeding beliefs and practices in infancy, found that maternal weight was not associated with neither concern about over- and underweight, using food to calm nor awareness of infant cues [44]. However, non-responsive feeding practices have shown to mediate the relationship between maternal and child obesogenic eating behaviors [74]. Given that maternal weight is a strong predictor for childhood obesity and maternal feeding practices are modifiable risk factors, more longitudinal studies are needed to disentangle the influence of maternal weight on maternal feeding styles and practices.

### Infant characteristics

#### Infant weight

Both the child´s weight and the mother´s concern about the child´s weight have in previous research been positively associated with maternal use of non-responsive feeding practices such as pressure to eat and restriction [20, 75–77]. Parents who consider their child as underweight may use pressure to make their child eat, while parents who perceive their child as overweight may restrict the child´s food intake. Parental use of these non-responsive feeding practices may negatively affect the child´s relationship with food [78] and undermine the child´s self-regulation of eating which in turn may lead to excessive weight gain [79].

In this study, infant weight was associated with maternal feeding styles and practices over time. Infant weight was associated with maternal concern over infant´s weight status, which is congruent with previous research using the IFQ [26, 30]. Mothers whose infants had lower BMI-for-age z-scores at five months of age were more concerned about their child being underweight and less concerned about their child being overweight and vice versa. We did not examine the association between maternal concern about under-/overweight at child age 5 months and maternal use of pressure and restriction at child age 24 months. However, our findings do suggest that infant weight at child age 5 months is associated with maternal use of non-responsive feeding practices at child age 12 and 24 months. Mothers of infants with a lower BMI-for-age z-scores at child age 5 months were more likely to have a low score on *Awareness of hunger and satiety cues* at child age 12 months. We found the same association at child age 5 months, although only almost significant (p = 0.051). We further found that mothers of infants with lower BMI-for-age z-scores at child age five months were more likely to have a high score on *Pressure* (CFQ) at child age 24 months. Previous longitudinal studies examining the direction of this relationship, have found that parents pressure their child to eat in response to the child´s weight rather than the reverse [80, 81]. Our results support this research, finding that lower infant weight at child age 5 months predicts a higher risk of maternal use of controlling feeding practices at child age 24 months.

#### Infant temperament

Temperament is a biologically based pattern of relatively stable individual characteristics present from birth [82]. Difficulties in conduct, self-regulation and temperament may lead to the development of problematic feeding via the disruptions this can cause to adaptive parent–child feeding interactions [83]. In the feeding situation, the infant´s cues of hunger and satiety may be more difficult to recognize and distinguish from other distress cues in a child with greater temperamental difficulties. This may in turn lead to more discordant responsiveness in feeding and less support of the child´s innate ability to regulate food intake [10]. Child temperament may further have a bidirectional relationship with feeding practices, where child negative affect put children at increased risk for the cascading relation between emotional feeding and emotional eating [38]. There is now emerging evidence for positive associations between infant temperament characterized by poorer self-regulation and self-soothing ability and later elevated adiposity indices [84].

We found that associations between infant temperament and maternal feeding beliefs and practices were prominent at 5 months of age. Mothers of infants with a “fussier” temperament reported higher concern about over- and underweight, less awareness of hunger and satiety cues and more use of food to calm. A more “difficult” infant temperament at child age 5 months was still associated with lower maternal awareness of hunger and satiety cues at child age 12 months. At child age 24 months, the association between infant temperament and the factor *Emotional feeding* (PFSQ) was almost significant (p = 0.06).

Our findings are in line with previous systematic reviews finding that parental perceived “difficult” child temperament influences parental feeding behavior [23, 85]. A cross-sectional study of Australian mother-infant dyads found that mothers of children with more difficult temperament reported lower awareness of infant cues, were more likely to use food to calm and reported higher concern over child weight [24]. Our study of Norwegian mother-infant dyads replicates these findings and underlines that associations between maternal perceptions about child temperament and maternal feeding practices are evident from infancy. Our results may indicate that perceived fussy temperament during infancy could be associated with maternal use of non-responsive feeding styles and practices also in a longer term. However, one should recognize that the child’s temperament may be a stable factor over time and thus a possible underlying cause of our findings.

#### Infant feeding mode

Caregiver sensitivity is fundamental during feeding to recognize and respond to the infant’s cues of internal hunger and satiation, but the ability of the caregiver to be attuned during feeding may be influenced by early feeding modality. A systematic review that examined associations between feeding mode and maternal responsiveness in feeding, found that cross-sectional observational studies consistently reported greater responsiveness among breastfeeding mothers than among formula-/bottle-feeding mothers. Longitudinal studies showed that longer breastfeeding duration predicted lower use of nonresponsive feeding practices during later childhood [86]. Also, infant eating behavior may be influenced by feeding mode. Some studies have shown that breastfeeding is associated with better child-eating self-regulation [87] and satiety-responsiveness [88]. In addition, infants who were breastfed have been found to show more engagement and disengagement cues during meals than formula-fed infants, perhaps making the breastfed infants easier to “read” [89].

Our results replicate previous research, finding that no breastfeeding is positively related to a non-responsive feeding style both in infancy and early childhood. At child age 5 months, infants who were not breastfed were more likely to have mothers who used food to calm. At child age 24 months, mothers of infants who were not breastfed at child age five months were more likely to have a high score on *Emotional Feeding* (PFSQ). It is theorized that the inability of a breastfeeding mother to assess infant consumption may promote greater confidence in the infant’s own ability to self-regulate intake and feed in response to satiation cues [10, 90]. However, studies specifically designed to assess causal mechanisms underlying associations between feeding mode and maternal responsiveness are needed to illuminate whether breastfeeding leads to responsive mothers or responsive mothers choose to breastfeed [86].

We further found that infants who were not breastfed at child age five months were less likely to have mothers with a high score on *Concerned about underweight* at child age 12 months. This is an isolated finding that may be difficult to interpret. A potential explanation could be that bottle-feeding and greater ability to assess and control how much the infant consumes, facilitates maternal perceived control regarding infant food-intake and weight development. Greater maternal perceived control may in turn lead to less concern. Nevertheless, this remains a speculation and should not be given significance.

### Strengths and limitations

Our study has some important strengths. We have investigated infant and maternal predictors of maternal feeding styles and practices in infancy and toddlerhood concurrently, including a wide range of both maternal and infant correlates aiming to reduce the risk of unmeasured confounding factors of importance. The use of well-known and validated instruments for the outcomes is a strength, and the longitudinal design of the study contributes with important knowledge regarding stability and continuity of early maternal feeding styles and practices. Our results align with previous research, which supports our findings.

The low variability in the socioeconomic status of the participating mothers is a weakness of this study. The mothers volunteered for participation, which is known to introduce a skewness towards a more well-educated population [91]. This may have resulted in an underestimation of socioeconomically patterned characteristics and could make it difficult to generalize from our findings. Further, the use of self-reported data may reduce the reliability of our findings.

Different instruments were used to map maternal feeding styles and practices due to the increasing age of the child, which increases the reliability of the assessment. However, this is a methodological weakness of the study and makes direct comparison over time more difficult. The cohort-specific cut-offs in the outcome variables may make the size-estimates difficult to reproduce in other studies but should not prevent reproducing the associations as such. Since the cut-off in all outcome variables were data-driven and thus arbitrary and cohort-specific, individual effect estimates should be interpreted with caution and considered indicative of an association rather than as precise measures. Given the number of relationships examined, the risk of Type I errors should be acknowledged. The statistically driven nature of the modelling could be a limitation as small numbers of observations in the models may yield results that fit poorly to the overall population. Lastly, the paper could be strengthened by using a more advanced statistical model and include interaction terms to investigate the relationship between maternal and child correlates further. A subsequent study with preplanned hypotheses is needed to confirm the observed associations.

## Conclusion

This is one of few studies to explore how maternal use of feeding styles and practices differs according to key mother and child correlates in infancy and to which extent these associations persist during the child´s first two years. Our findings confirm that maternal feeding styles and practices in infancy are associated with both maternal and child characteristics, and that some of these associations are sustained over time. This applies for maternal education and mental health symptoms as well as for infant weight, temperament and feeding mode. While infant temperament seemed to be of particularly importance in the first year of life, infant weight and feeding mode as well as maternal mental health symptoms during infancy continued to be associated with maternal responsiveness in feeding at 2 years of age. Our findings indicate that risk-factors that may have long-term implications for child weight and health outcomes can be identified early. Understanding modifiable early mother-child feeding patterns with potential influence on later eating behaviors in children, is important and have implications for clinical practice, public health nutrition actions and future research. Larger, population-based studies with longitudinal designs are needed to further explore these pathways.

## Supporting information

S1 Dataset (SAV)
